# Assessing the role of robotic proctectomy in obese patients: a contemporary NSQIP analysis

**DOI:** 10.1007/s11701-022-01380-2

**Published:** 2022-02-11

**Authors:** Alexa C. Glencer, Joseph A. Lin, Karen Trang, Anya Greenberg, Kimberly S. Kirkwood, Mohamed Abdelgadir Adam, Ankit Sarin

**Affiliations:** 1 Department of Surgery, University of California, San Francisco, CA USA; 2 School of Medicine, University of California, San Francisco, CA USA; 3 Colon and Rectal Surgery, Bakar Precision Cancer Medicine Building, University of California San Francisco, 1825 4th Street, 4th floor, San Francisco, CA 94158 USA

**Keywords:** Robotic proctectomy, Obese patients, Conversion, NSQIP

## Abstract

Robotic proctectomy has become increasingly popular for both benign and malignant indications. The purpose of this study was to determine if the robotic approach has a distinct advantage over laparoscopy in obese patients, which has been suggested by previous subgroup analyses. We performed a retrospective review of 2016–2018 National Surgery Quality Improvement Program (NSQIP) data to compare outcomes between patients who underwent robotic versus laparoscopic proctectomy, stratified by Body Mass Index (BMI) subgroups. We also compared outcomes of converted minimally invasive proctectomy to planned open operations. Four thousand four hundred eighteen (69.3%) patients underwent laparoscopic proctectomy, and 1956 (30.7%) patients underwent robotic proctectomy. Robotic proctectomy was associated with a significantly lower conversion rate compared to laparoscopic proctectomy (5.1% vs 12.3%; *p* = 0.002), and this relationship was maintained on an adjusted model. Obese (BMI > 30) patients were more likely to require conversion in both laparoscopic and robotic groups with the greatest difference in the conversion rate in the obese subgroup. Patients who underwent conversion had higher composite morbidity compared to patients who underwent planned open operations (50.8% vs 41.3%; *p* < 0.001). And among patients with rectal cancer, robotic proctectomy was associated with a greater incidence of positive radial tumor margins compared to laparoscopic proctectomy (8.0% vs 6.4%; *p* = 0.039), driven primarily by the obese subgroup. Our study demonstrates that robotic proctectomy is associated with a 7% lower conversion rate compared to laparoscopy and that obese patients are more likely to require conversion than non-obese patients. Among obese patients with rectal cancer, we identified an increased risk of positive radial margins with robotic compared to laparoscopic proctectomy.

**Supplementary Information:**

The online version contains supplementary material available at 10.1007/s11701-022-01380-2.

## Introduction

Robotic proctectomy has become an increasingly popular approach for rectal surgery. Compared with traditional laparoscopy, robotic assistance offers high-definition 3D vision, improved surgeon ergonomics, and a greater range of motion with instrument wrist articulation. This theoretically results in more precise and dexterous movements, particularly relevant in pelvic surgery given the deep and narrow working space. Patients associate robotics with smaller incisions, reduced infections, and greater precision [1].) The demand for robotic surgery continues to increase, and with it, the importance of understanding its impact on outcomes in different patient populations.

In previous studies, robotic proctectomy, as part of either low anterior resection (LAR) or abdominoperineal resection (APR), has been associated with decreased conversion to open surgery and decreased hospital length of stay (LOS) compared to laparoscopy [2]. However, it has also been associated with increased operative duration and as much as a 31% increased cost compared to open or laparoscopic proctectomy [3]. Therefore, controversy persists regarding the value of robotics. Given this controversy, it is imperative to study the impact of robotic surgery in specific patient populations to understand its differential impact and derive value through careful patient selection.

Obese patients are a specific population that may particularly benefit from the advantages of the robotic platform given the ability of the robot to offload the cognitive load of exposure from the surgeon to the robot, which would allow the surgeon to direct greater focus to precise dissection, resection, and reconstruction. This is especially important in the pelvis where exposure is challenging with any approach. As a result, robotic proctectomy in obese patients may lead to improved intraoperative and post-operative outcomes, potentially justifying increased costs and operative times. Previous BMI-based subgroup analyses suggested that decreased conversion associated with robotic proctectomy is meaningful in overweight and obese patients, but these analyses have been underpowered to draw definitive conclusions [4]. Studies that investigated robotic proctectomy in obese rectal cancer patients demonstrated marginal benefits in post-operative morbidity, but these studies were limited by small sample size [5, 6]. Thus, evidence supporting the role of robotic proctectomy for obese patients remains incomplete. We, therefore, sought to compare outcomes of robotic versus laparoscopic proctectomy, focusing on overweight and obese patients using a large, contemporary, and nationally representative dataset.

## Materials and methods

A retrospective analysis of the American College of Surgeons National Surgical Quality Improvement Program (NSQIP) database was conducted using 2016–2018 participant and targeted proctectomy data. NSQIP data are prospectively collected and validated by trained surgical clinical reviewers. We included patients ≥ 18 years old who underwent elective robotic or laparoscopic proctectomy, which included LAR and APR. We included patients who underwent open proctectomy as a single analysis comparing outcomes in this group to those among patients who had been converted from a minimally invasive approach. This study was approved by the Institutional Review Board of the University of California, San Francisco (UCSF): study number 18–26,677.

### Preoperative and operative characteristics

Preoperative characteristics included age, sex, race/ethnicity, body mass index (BMI), American Society of Anesthesiology (ASA) class, functional status, and diagnosis [cancer, benign tumor, diverticular disease, inflammatory bowel disease (IBD), vascular, and other]. BMI was categorized as underweight (< 18.5), normal weight (18.5–25), overweight (25–30), obese (30–40), and morbidly obese (> 40) according to Centers for Disease Control and Prevention (CDC) guidelines. Comorbidities included hypertension, diabetes (requiring hypoglycemic medications or insulin), smoking status, congestive heart failure (CHF), chronic obstructive pulmonary disease (COPD), renal failure, bleeding disorder, and chronic steroid use. Proctectomy-specific variables included prior chemotherapy, prior radiation therapy, clinical tumor stage, and location in the rectum for rectal cancer. Complications present before surgery (sepsis/septic shock; superficial, deep, and organ space surgical site infections [SSI]; ventilator dependence; pneumonia; urinary tract infection [UTI]) were included as predictors and used to adjust post-operative complications as defined by NSQIP.

Operative factors included the approach (open, laparoscopic, or robotic), wound class, and whether a blood transfusion (intraoperative or within 72 h of surgery) was received. For rectal cancer patients, clinical T stage and tumor location (upper third [> 10 cm from anal verge], middle third [5–10 cm from anal verge], and lower third [< 5 cm from anal verge]) were also included.

### Outcomes

The primary outcome was unplanned conversion to open surgery. Secondary outcomes included case duration, intraoperative or post-operative blood transfusion, LOS in cases without post-operative complications, unplanned readmission, unplanned reoperation, death within 30 days, anastomotic leak, ileus (determined by prolonged NPO status or need for nasogastric tube placement post-operatively), SSI, sepsis/ septic shock, wound dehiscence, pneumonia, reintubation or failure to wean from ventilator > 48 h, pulmonary embolism, deep vein thrombosis (DVT), acute renal insufficiency, UTI, *Clostridium difficile* infection, stroke, cardiac arrest, and myocardial infarction. All anastomotic leaks were included as organ space SSIs, but they were also separately counted based on positive identification on imaging (presence of extraluminal air–fluid levels or leakage of enteric contrast at the anastomosis) or specific notation by the surgeon. A composite morbidity outcome was created based on the occurrence of any of these complications. Margin status (distal and radial) was assessed for rectal cancer patients.

Secondary outcomes were compared between robotic and laparoscopic proctectomies using intention-to-treat with regard to approach: converted cases were categorized as the intended minimally invasive approach. Additionally, secondary outcomes were compared between patients who underwent either robotic or laparoscopic proctectomy converted to open, collectively termed minimally invasive converted to open, and planned open proctectomy.

### Statistical methods

Patient characteristics and post-operative outcomes were compared between the laparoscopic and robotic cohorts using t test for continuous variables and the Chi-square test for categorical data. Multivariable logistic models were created for all categorical outcomes using all available covariates without model selection or training sets. Covariates included year of surgery, primary diagnosis, age category, sex, race/ethnicity, BMI category, smoking status, ASA class, functional status, diabetes, hypertension requiring medications, chronic obstructive pulmonary disease (COPD), congestive heart failure (CHF), renal failure/dialysis, presence of ascites, distant cancer, steroid use, recent weight loss, bleeding disorder, preoperative transfusion, preoperative sepsis or ventilator, tumor location, neoadjuvant chemotherapy or radiation, and wound class. Linear, Gamma log-linked, Poisson, and log-transformed multivariable models to evaluate operative time and LOS were compared to account for right-skewness. Interaction terms between variables of interest were added to models based on clinical context. Subgroup regression analyses were performed by BMI category, diagnosis, and conversion status. Multicollinearity in regression models was assessed using generalized variance inflation factors. Missing categorical data were treated as separate categories. Analysis was performed using R version 4.0.2 (R Foundation for Statistical Computing).

## Results

### Demographic and preoperative characteristics

A total of 6374 minimally invasive (MIS) proctectomies were recorded in the NSQIP database from 2016 to 2018 with 4418 (69.3%) performed laparoscopically and 1956 (30.7%) performed robotically. Patients who underwent robotic proctectomy tended to be older and were more likely to be male (Table 1). Overweight, obese, and morbidly obese patients were all significantly more likely to undergo robotic compared to laparoscopic proctectomy.

**Table 1:** Demographic characteristics of patients undergoing MIS proctectomies, 2016–2018

Characteristic	Laparoscopic *n* (%)	Robotic *n* (%)	*p* Value
Age range (years)			
18–49	1593 (36.1)	597 (30.5)	** < 0.001**
50–59	1009 (22.8)	476 (24.3)	
60–69	958 (21.7)	522 (26.7)	
70 +	837 (18.9)	356 (18.2)	
Sex			
Female	2083 (47.1)	800 (40.9)	** < 0.001**
Male	2335 (52.9)	1156 (59.1)	
Race			
American Indian/Alaska Native	17 (0.4)	8 (0.4)	** < 0.001**
Asian	184 (4.2)	135 (6.9)	
Black/African American	188 (4.3)	129 (6.6)	
Native Hawaiian/Pacific Islander	8 (0.2)	6 (0.3)	
White	3065 (69.4)	1550 (79.2)	
Unknown	956 (21.6)	128 (6.5)	
Ethnicity			
Hispanic	207 (4.7)	140 (7.2)	** < 0.001**
Non-Hispanic	3363 (76.1)	1803 (89.5)	
Unknown	848 (19.2)	65 (3.3)	
BMI			
Underweight (BMI < 18.5)	158 (3.6)	59 (3.0)	**0.002**
Normal (BMI 18.5–24.9)	1597 (36.1)	626 (32.0)	
Overweight (BMI 25.0–29.9)	1418 (32.1)	648 (33.1)	
Obese (BMI 30.0–39.9)	1072 (24.3)	543 (27.8)	
Morbidly obese (BMI 40 +)	153 (3.5)	77 (3.9)	

*p*-values below 0.05 were considered statistically significant are indicated in bold

While patients with cancer represented the majority of patients overall, they were overrepresented in the robotic group (67.7% robotic vs 51.7% laparoscopic). Conversely, patients with inflammatory bowel disease were underrepresented in the robotic cohort (34.0% laparoscopic vs 19.2% robotic) as were patients on chronic steroids (16.8% laparoscopic vs 8.8% robotic; p < 0.001). Patients who underwent robotic proctectomy were more likely to have a higher ASA classification and to have hypertension requiring medication at baseline. Patients who underwent laparoscopic proctectomy for rectal cancer were more likely to have high rectal tumors (10–15 cm from the anal verge), whereas those who underwent robotic proctectomy for rectal cancer were more likely to have low (0–5 cm from the anal verge) or middle (5–10 cm from the anal verge) rectal tumors (Table 2).

**Table 2:** Preoperative characteristics of patients undergoing MIS proctectomies, 2016–2018

Characteristic	Laparoscopic *n* (%)	Robotic *n* (%)	*p* Value
Diagnosis			
Benign tumor	141 (3.0)	53 (2.6)	** < 0.001**
Cancer	2436 (51.7)	1362 (67.7)	
Inflammatory bowel disease	1601 (34.0)	386 (19.2)	
Prolapse	203 (4.3)	106 (5.3)	
Other	330 (7.0)	104 (5.2)	
ASA classification			
1	90 (2.0)	31 (1.6)	**0.048**
2	2073 (46.9)	857 (43.8)	
3	2156 (48.8)	1024 (52.4)	
4	95 (2.2)	44 (2.2)	
Hypertension requiring meds	1393 (31.5)	706 (36.1)	** < 0.001**
History of CHF	12 (0.3)	8 (0.4)	0.5
History of COPD	106 (2.4)	48 (2.5)	1
Smoker	616 (13.9)	297 (15.2)	0.2
Steroid use	743 (16.8)	173 (8.8)	** < 0.001**
Diabetes			
No	3938 (89.1)	1717 (87.8)	0.2
Non-insulin-dependent diabetes	306 (6.9)	160 (8.2)	
Insulin-dependent diabetes	174 (3.9)	79 (4.0)	
Tumor location (if diagnosis = rectal cancer)			
Lower third	1000 (22.6)	690 (35.3)	** < 0.001**
Middle third	679 (15.4)	383 (19.6)	
Upper third	404 (9.1)	129 (6.6)	
Preoperative T stage (rectal cancer cases only)			
T1	107 (4.7)	65 (4.9)	0.002
T2	362 (15.9)	168 (12.7)	
T3	1124 (49.4)	652 (49.2)	
T4	153 (6.7)	127 (9.6)	
Unknown	530 (23.3)	312 (23.6)	
Preoperative N stage (rectal cancer cases only)			
N0	974 (56.2%)	575 (55.4%)	0.7
N1	557 (32.1%)	330 (31.8%)	
N2	202 (11.7%)	132 (12.7%)	
Preoperative M stage (rectal cancer cases only)			
M0/Mx	1361 (95.3%)	800 (93.7%)	0.1
M1	67 (4.7%)	54 (6.3%)	

*p*-values below 0.05 were considered statistically significant are indicated in bold

### Conversion to open

Robotic proctectomy was associated with a significantly lower rate of conversion to open compared to laparoscopic proctectomy (5.1% vs 12.3%; p = 0.001) (Table 3), and this relationship was maintained on an adjusted model (odds ratio (OR) 0.37 (95% confidence interval [CI] 0.29–0.46, p = 0.001). Men were more likely to require conversion compared to women (OR 1.55, CI 1.29–1.86; p < 0.001). Obese patients were also more likely to require conversion (OR 2.71, CI 2.14–3.42; p = 0.002 for BMI 30–40; OR 3.96, CI 2.66–5.90; p < 0.001 for BMI > 40). However, there was not a statistically significant interaction between male gender and obesity when modeling conversion nor was there a statistically significant interaction between male gender and minimally invasive approach that affected conversion. Older patients (> 60 years old), those with COPD, and those categorized as ASA class 3 or 4 were more likely to undergo conversion. A preoperative diagnosis of rectal cancer or IBD was not associated with conversion (Table 4).

**Table 3:** Outcomes associated with patients undergoing laparoscopic and robotic proctectomies

Outcome	Laparoscopic *n* = 4711	Robotic *n* = 2011	OR/linear coefficient^a^	95% CI	*p* Value
**Conversion to open**	**545 (12.3%)**	**99 (5.1%)**	**0.37**	**0.29–0.46**	**0.002**
**Mean case duration (mins)**	**289 ± 115**	**317 ± 128**	** + 21**	**15–28**	** < 0.001**
**LOS w/o complication (days)**	**5.0 ± 3.1**	**4.6 ± 2.6**	**− 0.2**	**− 0.4 to − 0.02**	**0.02**
Readmission	689 (15.6%)	313 (16.0%)	1.08	0.92–1.26	0.3
Unplanned reoperation	237 (5.6%)	112 (5.7%)	1.11	0.86–1.43	0.4
Death within 30 days	16 (0.4%)	4 (0.2%)	0.46	0.13–1.64	0.2
Composite morbidity	1480 (33.5%)	589 (30.1%)	0.98	0.87–1.12	0.8
Anastomotic leak	120 (2.7%)	53 (2.7%)	1.35	0.94–1.95	0.10
Ileus	787 (17.8%)	289 (14.8%)	0.89	0.76–1.04	0.14
Transfusion (< 72 h post-op)	308 (7.0%)	114 (5.8%)	0.86	0.68–1.10	0.2
Organ space SSI	303 (6.9%)	133 (6.8%)	1.23	0.98–1.56	0.08
Superficial SSI	171 (3.9%)	68 (3.5%)	1.08	0.79–1.48	0.6
**Wound dehiscence**	**44 (1.0%)**	**23 (1.2%)**	**1.71**	**1.01–2.91**	**0.047**
Sepsis	133 (3.0%)	53 (2.7%)	1.01	0.71–1.44	0.9
Urinary tract infection	143 (3.2%)	60 (3.1%)	1.16	0.83–1.62	0.4
**Pneumonia**	**62 (1.4%)**	**12 (0.6%)**	**0.49**	**0.25–0.96**	**0.036**
Pulmonary embolus	18 (0.4%)	5 (0.3%)	0.60	0.21–1.70	0.3
**Deep vein thrombosis**	**67 (1.5%)**	**12 (0.6%)**	**0.48**	**0.25–0.90**	**0.02**
**Acute renal insufficiency**	**50 (1.1%)**	**13 (0.7%)**	**0.46**	**0.24–0.89**	**0.02**
Myocardial infarction	17 (0.4%)	8 (0.4%)	1.23	0.46–3.28	0.7
*Clostridium difficile* infection	22 (0.5%)	7 (0.4%)	0.83	0.34–2.05	0.7
Oncologic outcomes for rectal cancer patients					
**Positive radial margin**	**136 (6.4%)**	**95 (8.0%)**	**1.38**	**1.02–1.88**	**0.039**
Positive distal margin	43 (2.1%)	22 (1.8%)	0.98	0.54–1.75	0.9
Lymph-node yield		Laparoscopic	Robotic	*p* value	
< 12 lymph nodes		797 (18.0%)	425 (21.7%)	0.6	
12 or more lymph nodes		2159 (48.9%)	1103 (56.4%)		
Pathologic T stage					
T1		486 (21.8%)	296 (23.2%)	0.3	
T2		614 (27.6%)	371 (29.1%)		
T3		998 (44.8%)	528 (41.4%)		
T4		130 (5.8%)	80 (6.3%)		
Pathologic N stage					
N0		1463 (65.9%)	846 (66.7%)	0.9	
N1		558 (25.1%)	308 (24.3%)		
N2		198 (8.9%)	114 (9.0%)		
Pathologic M stage					
M0		1536 (95.6%)	937 (95.3%)	0.8	
M1		70 (4.4%)	46 (4.7%)		

*p*-values below 0.05 were considered statistically significant are indicated in bold

^a^Adjusted by year of surgery, age, sex, race, ethnicity, body mass index, diagnosis, American Society of Anesthesiologists classification, smoking, functional status, diabetes, hypertension requiring medications, congestive heart failure, chronic obstructive pulmonary disease, bleeding disorder, steroids, recent transfusion, preoperative sepsis, tumor location, wound classification, and prior chemotherapy or radiation. Additionally, wound closure was included as a predictor for superficial SSI, dehiscence, and sepsis

**Table 4:** Preoperative characteristics of converted vs non-converted MIS proctectomy patients

Characteristic	Remained MIS *n* (%)	MIS converted to open *n* (%)	*p* Value
Year			
2016	1719 (30.0)	210 (32.6)	0.2
2017	1983 (34.6)	227 (35.2)	
2018	2028 (35.5)	207 (32.1)	
Diagnosis			
Benign	172 (3.0)	19 (3.0)	**0.006**
Cancer	3288 (57.4)	390 (57.0)	
Inflammatory bowel disease	1670 (29.1)	189 (29.3)	
Prolapse	273 (4.8)	15 (2.3)	
Other	327 (5.7)	54 (8.4)	
ASA category			
1	113 (2.0)	8 (1.2)	**0.01**
2	2650 (46.2)	280 (43.5)	
3	2846 (49.7)	334 (51.9)	
4	119 (2.1)	20 (3.1)	
Age category (years)			
18–49	1998 (34.9)	192 (29.8)	**0.13**
50–59	1331 (23.2)	154 (23.9)	
60–69	1319 (23.0)	161 (25.0)	
70 +	1059 (18.5)	134 (20.8)	
Male gender	3085 (53.8)	406 (63.0)	** < 0.001**
Hypertension requiring meds	1822 (31.8)	277 (43.0)	** < 0.001**
History of CHF	20 (0.3)	0	0.3
History of COPD	130 (2.3)	24 (3.7)	**0.03**
Smoker	811 (14.2)	102 (15.8)	0.27
Steroid use	831 (14.5)	85 (13.2)	0.4
Diabetes			
No	5104 (89.1)	595 (85.6)	0.03
Non-insulin-dependent diabetes	406 (7.1)	60 (9.3)	
Insulin-dependent diabetes	220 (3.8)	33 (5.1)	
Tumor location (if diagnosis = rectal cancer)			
Lower third	1555 (27.1)	135 (21.0)	** < 0.001**
Middle third	946 (16.5)	116 (18.0)	
Upper third	457 (8.0)	76 (11.8)	

*p*-values below 0.05 were considered statistically significant are indicated in bold

When comparing the profiles of patients who required conversion from robotic surgery to patients who required conversion from laparoscopic surgery, there were no significant differences in demographic variables, BMI distribution, comorbidity profiles, or preoperative diagnosis between the two groups. Conversion rates were lower for the robotic approach compared to the laparoscopic approach across all BMI subgroups, but the difference was greater in the obese subgroup (10.9%) and morbidly obese subgroup (8.6%) than in either the overweight (6.6%) or normal weight (5.6%) subgroups (Supplemental Table 1).

Compared to patients who underwent planned open operations, patients who underwent conversion to open from a minimally invasive approach had longer operations (335 min vs 248 min; p < 0.001) and greater composite morbidity (50.8% vs 41.3%; p < 0.001). The higher composite morbidity for converted patients was driven by higher rates of organ space SSI (10.2% vs 6.8%; p = 0.001), anastomotic leak (4.6% vs 3.1%; p = 0.049), ileus (25.3% vs 19.2%; p < 0.001), and acute renal insufficiency (2.0% vs 1.0%; p = 0.023) (Table 5).

**Table 5:** Outcomes for planned open vs converted MIS proctectomies

Outcome	Planned open *n* = 6260	MIS converted to open *n* = 693	*p* Value
**Mean case duration (mins)**	**248**	**335**	** < 0.001**
LOS w/o complication (days)	5.5	6.0	0.11
Readmission	923 (14.7%)	111 (16.0%)	0.40
Unplanned reoperation	403 (6.4%)	49 (7.1%)	0.58
Positive radial margin (cancer)	260 (11.3%)	33 (9.1%)	0.26
Positive distal margin (cancer)	74 (3.2%)	9 (2.5%)	0.60
Death within 30 days	87 (1.4%)	5 (0.7%)	0.199
**Composite morbidity**	**2587 (41.3%)**	**352 (50.8%)**	** < 0.001**
**Ileus**	**1205 (19.2%)**	**175 (25.3%)**	** < 0.001**
Transfusion (72 h post-op)	1011 (16.2%)	123 (17.7%)	0.305
**Anastomotic leak**	**196 (3.1%)**	**32 (4.6%)**	**0.049**
**Organ space SSI**	**424 (6.8%)**	**71 (10.2%)**	**0.001**
Superficial SSI	401 (6.4%)	50 (7.2%)	0.46
Wound dehiscence	118 (1.9)	11 (1.6)	0.687
Sepsis	213 (3.4)	28 (4.0)	0.446
Septic shock	81 (1.3)	15 (2.2)	0.091
Urinary tract infection (UTI)	226 (3.6)	30 (4.3)	0.397
Pneumonia	131 (2.1)	14 (2.0)	1
Pulmonary embolus (PE)	72 (1.2)	13 (1.9)	0.142
Deep vein thrombosis (DVT)	74 (1.2)	10 (1.4)	0.679
**Acute renal insufficiency**	**62 (1.0)**	**14 (2.0)**	**0.023**
Myocardial infarction	46 (0.7)	5 (0.7)	1
*Clostridium difficile* infection	45 (0.7)	2 (0.3)	0.286

*p*-values below 0.05 were considered statistically significant are indicated in bold

### Surgical complications and post-operative morbidity

There was no difference in mortality or composite morbidity between the robotic and laparoscopic groups; however, some complications occurred at significantly different rates between the two approaches (Table 3). The mean duration of robotic cases was significantly longer than that of laparoscopic cases (317 min vs 289 min; p < 0.001) (Table 3). When adjusted for all covariates, the robotic approach remained associated with a significantly longer operative time (+ 21 min, 95% CI 15–28, p < 0.001). There was a significant difference in hospital length of stay (LOS), pneumonia, deep vein thrombosis (DVT), and acute renal insufficiency favoring the robotic group. There was no difference in the incidence of organ space surgical site infection (SSI), anastomotic leak, ileus, pulmonary embolus, readmission, or unplanned reoperation between the two groups. There was a small but statistically significant difference in wound dehiscence favoring the laparoscopic group (Table 3).

### Oncologic outcomes for patients with rectal cancer

Patients with rectal cancer were more likely to have positive radial tumor margins if they underwent robotic rather than laparoscopic proctectomy (8.0% vs 6.4%, respectively; adjusted OR 1.38, 95% CI 1.02–1.88; *p* = 0.039) (Table 3). When subdivided by BMI category, this trend toward increased risk of radial margin positivity for the robotic approach was seen primarily for obese (BMI 30–40) patients (adjusted OR 1.98, 95% CI 1.0–3.94; *p* = 0.051). These results were adjusted for tumor T stage and location relative to the anal verge, as low rectal tumors (0–5 cm from the anal verge) and mid-rectal tumors (5–10 cm from the anal verge) were associated with significantly increased risk of positive radial margins (low rectal tumors adjusted OR 2.62, 95% CI 1.51–4.55, *p* = 0.001; mid-rectal tumors adjusted OR 1.66, 95% CI 0.92–3.00, *p* = 0.09). Neoadjuvant radiation therapy was associated with a significantly decreased risk of positive radial margins (adjusted OR 0.55, 95% CI 0.41–0.73, *p* < 0.001).

## Discussion

Robotic proctectomies represented 30% of our cohort, which aligns more closely with modern practice patterns compared to previously studied cohorts in which only 10% of patients underwent robotic proctectomy.(7) This study is also larger than any other contemporary analysis of robotic proctectomy patients [2]. Given the rapid uptake and expansion of robotic proctectomy, detailed contemporary analysis of its associated morbidity and value are essential. Furthermore, given the size of our cohort, we were able to evaluate outcomes in BMI-based subgroups.

Our study demonstrates that patients undergoing robotic compared to laparoscopic proctectomy were more likely to be older, male, obese, and have higher ASA class. Consistent with prior studies, robotic proctectomy was associated with increased operative time in our cohort [2, 8, 9]. However, the mean operative time for robotic proctectomy in our cohort was only 21 min longer than the mean operative time for laparoscopic proctectomy when adjusted for covariates. The decreased difference in mean operative time between minimally invasive approaches using contemporary data suggests that proficiency in performing robotic proctectomies may be increasing and that the difference in operative time compared to the laparoscopic approach may become clinically insignificant over time.

We demonstrated a significant difference in the conversion rate of robotic compared to laparoscopic proctectomy (5.1% vs 12.3%, respectively), an advantage maintained after multivariable adjustment. This reaffirms findings from other proctectomy-specific studies and aligns with recent conversion rates reported [2, 7, 9, 10]. Of note, the ROLARR randomized trial did not find a significant difference in conversion rates between robotic and laparoscopic surgery for patients with rectal cancer; however, its results likely no longer reflect current practice patterns as enrollment closed in 2014 and participating surgeons had far greater experience using the laparoscopic rather than the robotic platform [11]. Patients converted to open surgery in our study were more likely to be male, obese, older, and have more comorbidities. As these are the same factors that were more common among patients undergoing the robotic approach, it suggests that surgeons were selecting patients for the robotic approach who were at greatest risk of conversion and that, despite this selection, multivariate analysis still demonstrated that the conversion rate was significantly lower in patients undergoing robotic proctectomy.

In our study, the significantly lower conversion rate of robotic compared to laparoscopic proctectomy was maintained in normal, overweight, and obese subgroups; however, the difference in the conversion rate favoring the robotic approach was greater for the obese subgroup (10.9%) than for either the overweight (6.6%) or normal weight (5.6%) subgroups. This suggests that obese patients derive particular benefit from robotic compared to laparoscopic proctectomy, perhaps due to the weight of an obese abdominal wall being offloaded on the robot rather than on the surgeon. The lower conversion rate conferred by robotic surgery allows more medically complex patients to successfully undergo minimally invasive surgery. It also likely explains the significantly lower LOS in patients undergoing a robotic approach, and may explain the differences in post-operative pneumonia, DVT, and acute renal insufficiency favoring the robotic group.

Surgeons may choose a minimally invasive approach without regard to the likelihood of success, planning to convert to open if needed, with the rationale that conversion to an open operation poses the same risks as a planned open operation. However, we found that conversion has consequences. In comparing patients who underwent conversion from a minimally invasive approach to patients who underwent planned open surgery using an intention-to-treat analysis, converted patients had significantly increased composite morbidity. This increased composite morbidity is multifactorial but appears largely driven by increased rates of organ space surgical site infections, at least in part due to increased anastomotic leak rates. Patients undergoing proctectomy require thoughtful and individualized operative planning with consideration of the comorbid or anatomical factors that may increase risk of conversion and careful selection for planned open surgery. When a minimally invasive approach is considered for obese patients in particular, the robotic platform may increase the likelihood of success.

Use of the robotic approach for rectal cancer operations has continued to grow. Given that laparoscopic proctectomy failed to reach non-inferiority compared to open proctectomy for pathologic outcomes in rectal cancer, as demonstrated by the ACOSOG Z6051 trial [12], and that robotic proctectomy has not been directly compared to the open approach, ongoing analysis of outcomes for robotic rectal cancer surgery is imperative. Several studies have demonstrated no difference in oncologic outcomes between robotic and laparoscopic proctectomy for rectal cancer, but these did not analyze outcomes by BMI subgroup [9, 13–16].

In our study, when we examined proctectomies done for rectal cancer in obese patients (controlling for T stage), we identified a significantly increased risk of positive radial margins among patients who underwent robotic versus laparoscopic proctectomy. This could be the result of the preferred use of the robotic approach for lower rectal tumors, especially in this cohort of patients. In obese patients, mesorectal adipose tissue obscures natural tissue planes and can make an adequate dissection in the deep pelvis particularly challenging. It may also be that the most difficult cases are performed at tertiary-care referral centers that are likely to have robotic capabilities. As the robotic approach becomes more popular, comparing oncologic outcomes to the gold standard of open proctectomy in obese patients will be essential [13, 15]. Unfortunately, our analysis is limited to 30-day post-operative outcomes and an oncologic analysis would be best addressed with longer term data not available in NSQIP.

In addition to lack of long-term oncologic data, an important limitation of this study is the selection bias inherent in the nature of retrospective analyses and the clinical heterogeneity of our study population. While NSQIP data are rigorously collected by certified reviewers, it is limited to the specific variables being collected and excludes reporting hospital type, surgeon experience, use of diverting ostomy, and neoadjuvant chemotherapy regimen [17]. Especially with regard to conversions, the lack of granular data pertaining to the experience of the surgeon in performing laparoscopic or robotic proctectomy and the hospital type (academic, high-volume robotic institution, and community practice) limits interpretation of our results. While all surgeons performing robotic operations must receive training in the form of online modules, time spent on a practice counsel with tissue training, and in-person proctoring from Intuitive Surgical (manufacturer of the Da Vinci robotic system), there is variability in terms of the specialty training of the individual surgeon and prior robotic experience. We were also unable to exclude centers without robotic capabilities, as NSQIP does not indicate which included centers have surgical robots available; as a result, some of the laparoscopic cases were inevitably performed via this approach simply because a robotic approach was not an option. A prospective trial controlling for these variables is warranted to more definitively determine if there is benefit of the robotic approach relative to either the open or laparoscopic approach, particularly in obese patients at higher risk of surgical morbidity.

## Conclusion

This study provides an updated evaluation of the use of robotic versus laparoscopic proctectomy in a rapidly evolving surgical climate and assesses outcomes in BMI-based subgroups. Nearly 30% of proctectomies in our contemporary cohort were performed robotically with the greatest reduction in conversions compared to the laparoscopic approach in the obese subgroup. Unplanned conversion to open surgery was associated with increased composite morbidity compared to planned open proctectomy. And among obese patients with rectal cancer, we identified a significantly increased risk of positive radial margins following robotic compared to laparoscopic proctectomy. Overall, these data suggest that further study of the particular benefits and risks of robotic proctectomy in obese patients is warranted in a dedicated randomized trial.

## Supplementary Information

Below is the link to the electronic supplementary material.

Supplementary file1 (DOCX 25 KB)

Supplementary file2 (XLSX 14052 KB)

## Data Availability

Available with the corresponding author and will be presented on special request.
