# Supplementary material for: Assessing the role of robotic proctectomy in obese patients: a contemporary NSQIP analysis
Source: J Robot Surg. 2022 Feb 11;16(6):1391–9. doi: 10.1007/s11701-022-01380-2 (PMC9365884; doi:10.1007/s11701-022-01380-2)
Supplement: Supplementary file 1 — Supplementary file1 (DOCX 25 KB) [file 11701_2022_1380_MOESM1_ESM.docx]

**Supplemental Table 1.** Outcomes stratified by BMI subgroup

| **Outcome** | **Laparoscopic (n %)** | **Robotic**  **(n %)** | **p-value** | **OR/linear coefficient** | **95% CI** | **p-value** |
| --- | --- | --- | --- | --- | --- | --- |
| **Normal weight (BMI 18.5-25)** | | | | | | |
| **Conversion** | **129 (8.1)** | **14 (2.2)** | **<0.001** | **0.27** | **0.14 – 0.47** | **<0.001** |
| **OR time (mins)** | **267** | **288** | **<0.001** | **16.79** | **6.35 – 27.23** | **0.002** |
| **Length of stay (days)** | **4.9** | **4.5** | **0.013** | **-0.32** | **-0.61 – -0.03** | **0.029** |
| Reoperation | 88 (5.5) | 35 (5.6) | 1 | 1.02 | 0.65 – 1.57 | 0.9 |
| Readmission | 239 (15.0) | 90 (14.4) | 0.8 | 1.05 | 0.78 – 1.39 | 0.7 |
| Positive radial margin | 54 (7.8) | 27 (7.4) | 0.9 | 1.25 | 0.70 – 2.23 | 0.4 |
| Positive distal margin | 16 (2.3) | 3 (0.8) | 0.13 | 0.35 | 0.07 – 1.35 | 0.16 |
| **Overweight (BMI 25-30)** | | | | | | |
| **Conversion** | **160 (11.3)** | **31 (4.8)** | **<0.001** | **0.42** | **0.27 – 0.63** | **<0.001** |
| **OR time (mins)** | **292** | **321.67** | **<0.001** | **28.59** | **17.49 – 39.70** | **<0.001** |
| Length of stay (days) | 5.0 | 4.6 | 0.074 | -0.26 | -0.68 – 0.15 | 0.2 |
| Reoperation | 64 (4.5) | 39 (6.0) | 0.18 | 1.47 | 0.93 – 2.30 | 0.09 |
| Readmission | 223 (15.7) | 112 (17.3) | 0.4 | 1.13 | 0.86 – 1.48 | 0.34 |
| Positive radial margin | 46 (6.6) | 36 (8.9) | 0.2 | 1.31 | 0.77 – 2.21 | 0.3 |
| Positive distal margin | 15 (2.1) | 11 (2.7) | 0.7 | 1.15 | 0.43 – 3.02 | 0.8 |
| Obese (BMI 30-40) | | | | | | |
| **Conversion** | **202 (18.8)** | **41 (7.6)** | **<0.001** | **0.34** | **0.23 – 0.48** | **<0.001** |
| **OR time (mins)** | **318** | **340** | **0.001** | **16.58** | **3.31 – 29.85** | **0.014** |
| Length of stay (days) | 5.1 | 4.8 | 0.098 | -0.05 | -0.45 – 0.34 | 0.8 |
| Reoperation | 61 (5.7) | 29 (5.3) | 0.9 | 1.11 | 0.66 – 1.83 | 0.7 |
| Readmission | 172 (16.0) | 92 (16.9) | 0.7 | 1.16 | 0.85 – 1.57 | 0.4 |
| **Positive radial margin** | **27 (4.7)** | **26 (8.0)** | **0.065** | **2.21** | **1.12 – 4.46** | **0.024** |
| Positive distal margin | 9 (1.6) | 7 (2.1) | 0.8 | 1.75 | 0.50 – 6.57 | 0.4 |
| Morbidly obese (BMI > 40) | | | | | | |
| Conversion | 34 (22.2) | 11 (14.3) | 0.2 | 0.44 | 0.16 – 1.13 | 0.097 |
| **OR time (mins)** | 334 | 378 | 0.027 | 35.18 | -11.69 – 82.1 | 0.14 |
| **Length of stay (days)** | **5.6** | **4.4** | **0.003** | **-0.84** | **-1.67 – -0.01** | **0.048** |
| Reoperation | 15 (9.8) | 8 (10.4) | 1 | 0.46 | 0.08 – 2.29 | 0.4 |
| Readmission | 34 (22.2) | 14 (18.2) | 0.6 | 1.01 | 0.37 – 2.71 | 0.9 |
| Positive radial margin | 5 (5.8) | 2 (3.8) | 0.9 | Did not converge | | |
| Positive distal margin | 2 (2.4) | 1 (1.9) | 1.0 | Did not converge | | |
